# Point Defects in Silicon-Doped β-Ga_2_O_3_: Hybrid-DFT Calculations

**DOI:** 10.1021/acsomega.3c05557

**Published:** 2023-11-09

**Authors:** Asiyeh Shokri, Yevgen Melikhov, Yevgen Syryanyy, Iraida N. Demchenko

**Affiliations:** † Institute of Plasma Physics and Laser Microfusion, ul. Hery 23, 01-497 Warsaw, Poland; ‡ Institute of Fundamental Technological Research Polish Academy of Sciences, ul. Pawinskiego 5b, 02-106 Warsaw, Poland; § Institute of Microelectronics and Optoelectronics, Warsaw University of Technology, ul. Koszykowa 75, 00-662 Warsaw, Poland

## Abstract

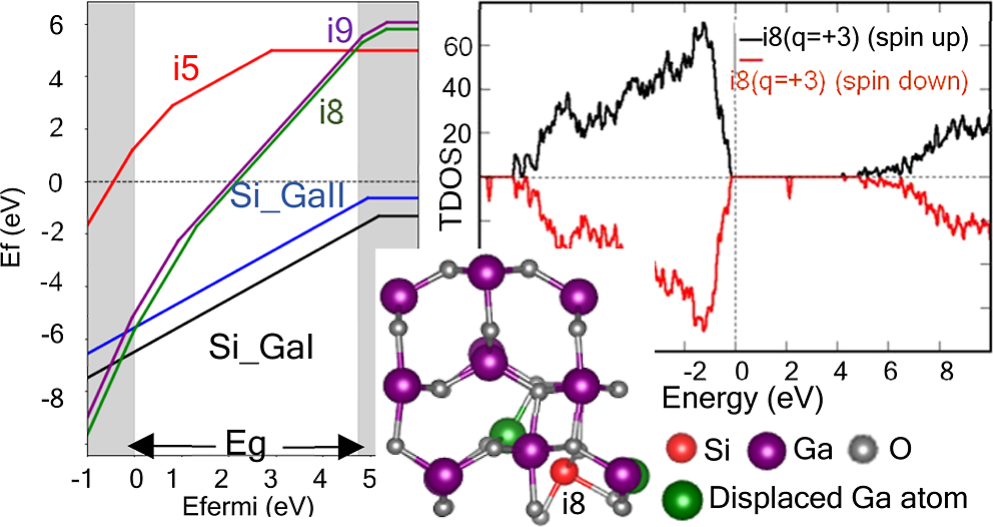

In this work, hybrid density functional theory calculations are used to evaluate the structural and electronic properties and formation energies of Si-doped β-Ga_2_O_3_. Overall, eight interstitial (Si_i_) and two substitutional (Si_Ga_) positions are considered. In general, our results indicate that the formation energy of such systems is significantly influenced by the charge state of the defect. It is confirmed that it is energetically more favorable for the substitution process to proceed under Ga-poor growth conditions than under Ga-rich growth conditions. Furthermore, it is confirmed that the formation of Si_GaI_ with a tetrahedral coordination geometry is more favorable than the formation of Si_GaII_ with an octahedral one. Out of all considered interstitial positions, due to the negative formation energy of the Si +3 charge state at i_8_ and i_9_ interstitial positions over the wide range of Fermi energy, this type of defect can be spontaneously stable. Finally, due to a local distortion caused by the presence of the interstitial atom as well as its charge state, these systems obtain a spin-polarized ground state with a noticeable magnetic moment.

## 1 Introduction

In recent years, the monoclinic phase of gallium oxide, known as β-Ga_2_O_3_, has gained significant attention and importance in various fields due to its exceptional properties and versatile applications. With a band gap value typically falling within the range of 4.5–4.9 eV,^1−4^ the thermally stable β-Ga_2_O_3_ phase holds great promise for revolutionizing diverse areas, ranging from electronics to optoelectronics. This phase has found particular significance in the development of cutting-edge devices, including Schottky barrier diodes,^5,6^ metal-oxide-semiconductor field-effect transistors (MOSFETs),^7^ metal–semiconductor field-effect transistors,^8^ solar-blind photodetectors,^9^ resistance random access memory devices,^10^ gas sensors,^11^ and spintronic devices.^12^ Furthermore, the remarkable properties and potential applications of the monoclinic phase of gallium oxide position it as a game-changing material in the field of power electronics, poised to revolutionize the power electronics industry.^7^

Defects in the crystal structure can significantly affect the performance and reliability of devices based on Ga_2_O_3_.^13^ In the monoclinic compound with low symmetry, several point defects need to be considered for vacancy and substitution studies, and in addition, interstitial sites, which can be occupied by neutral or charged atoms, must also be considered. According to the literature, some primary defects are considered to be electrically active. For example, gallium vacancies (*V*_Ga_), as well as their complexes with hydrogen, are deep acceptors, but gallium interstitial (with just one of the lowest formation energies the authors have found), Ga_i_,^14^ is a shallow donor. On the other hand, the oxygen vacancy and interstitial are expected to be deep donors and hence electrically neutral for Fermi-level positions close to the conduction band minimum (CBM).^14−16^

Dopants also play a crucial role in modifying the electrical and optical properties of the material, enabling the creation of specific device functionalities. By carefully selecting and controlling dopants, it is possible to tailor the conductivity type (n-type or p-type) and achieve the desired carrier concentration in gallium oxide. The ability to precisely dope β-Ga_2_O_3_ with impurities such as Sn, Si, Ge, and Mg enables the realization of high-quality n-type epitaxial films, offering a wide range of electron densities from 1 × 10^16^ to 1 × 10^19^ cm^–3^. Theoretical calculations indicate that Si, Ge, and Sn serve as the most common shallow donor impurities, with Si predicted to be the shallowest donor among them.^17^ Recent *ab initio* study of complexes of substitutional defects shows that doping Ga_2_O_3_ with Si could lead to an acceptor with Si_O_ coupled with H_Ga_ or to a donor with Si_Ga_.^18^ Despite extensive research on Si doping in β-Ga_2_O_3_ and the preference for Si substitution at the Ga_I_ site rather than at the interstitial positions,^15,16^ a comprehensive study of the various possible interstitial positions, their formation energy, and the replacement of intrinsic atoms as a result of interstitial doping is still lacking.

One of the reasons for the absence of such studies has been the understanding that in the case of Si interstitials, in a thermodynamically stable condition, only a small concentration of Si_i_ atoms would be present due to the negative formation energy of Si_Ga_.^18^ However, the situation could change dramatically when a suitable out-of-equilibrium growth technique, such as ion implantation doping, is used. In this case, due to the ballistic nature of this process, the buildup of a lattice disorder occurs and it is accompanied by the formation of various types of defects in relatively large concentrations. Recent experimental studies of β-Ga_2_O_3_:Si system prepared by ion implantation with Si ions show that Si interstitials occur in large concentration but not as isolated atoms but as complexes of Si_i_ with gallium and/or oxygen vacancies.^19^ Thus, to better understand the changes in the properties of β-Ga_2_O_3_ and gallium oxide-based materials, the most favorable interstitial sites should be identified through further analysis and simulations.

In the study by Blanco et al.,^20^ a comprehensive theoretical investigation of point defects in β-Ga_2_O_3_ was carried out, focusing on the ionic conductivity of the material. The energetics and diffusion properties of both the host lattice and dopant ions in β-Ga_2_O_3_ were examined within the framework of the shell model. Overall, eight different optimum configurations were identified for the Ga interstitials. Then, specifically for the Si dopant, the researchers adopted an ionic description considering Si^4+^. Such an approach assumes that the electronic distributions of positively charged ions do not undergo significant changes upon incorporation of ions into the lattice of β-Ga_2_O_3_. This simplification allowed for a more straightforward treatment of the dopant and facilitated the analysis of its behavior within the crystal structure.

In this article, we explore the doping properties of Si in the monoclinic phase of gallium oxide. Specifically, we investigate the formation energies of Si in eight interstitial sites and two distinct substitutional sites (Ga_I_ and Ga_II_). Our objective in this work is to analyze the relaxed positions and behavior of the Si dopants, calculate transition energies, and examine their impact on the electronic structure and optical properties of the pristine material.

## 2 Computational Methods

In this study, the first-principles density functional theory (DFT) calculations have been performed using the Vienna *ab initio* simulation package (VASP, v. 6.3.2) with projector augmented wave potentials.^21−24^ To accurately capture the structural and electronic properties of β-Ga_2_O_3_, the HSE06 hybrid functional^25^ was employed. This functional incorporates a fraction of exact exchange (0.32)^26^ and a fixed screening parameter of 0.2 Å^–1^. All calculations were spin polarized. The formation energy of an impurity with charge state *q* can be calculated using^27^
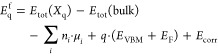
1Here, *E*_tot_(*X*_q_) represents the total energy of the supercell containing the defect in charge state *q*, and *E*_tot_(bulk) denotes the total energy of the defect-free crystal. *n*_*i*_ represents the change in the number of atoms due to the formation of a defect: it is negative if the atom of the *i*-th species is removed and positive in case an atom of the *i*-th species is added. The term *q*·(*E*_VBM_ + *E*_F_) accounts for the energy change upon the removal or addition of electrons during the formation of charged defects, with *E*_F_ representing the Fermi energy of β-Ga_2_O_3_ and *E*_VBM_ being the valence band maximum (VBM) value. To account for the elimination of false electrostatic interactions between charged defect supercells, the Freysoldt–Neugebauer–Van de Walle correction term, *E*_corr_, was included.^28^ The experimental value of 10 is taken for the dielectric constant.^29^ The chemical potentials μ_*i*_ of the elements calculated in this work are −5.69 eV for Si (from Si bulk), −3.38 eV for Ga (bulk Ga metal), and −7.187 eV for O (O_2_ molecules), enabling the determination of the Ga-rich (O-poor) and Ga-poor (O-rich) limits based on the enthalpy of formation of β-Ga_2_O_3_.

To construct the formation energy curve for a chosen defect, it is necessary to perform calculations according to eq 1 for a set of charge states *q* (typical values for *q* are 0, ±1, ±2, and ±3), considering the Fermi energy *E*_F_ as an independent variable in eq 1. Then, by varying *E*_F_, it is possible to identify the charge state q* that has the lowest formation energy *E*_q*_^f^ among all of the studied charges at that particular value of *E*_F_. By plotting values of *E*_q*_^f^ as a function of *E*_F_, the formation energy curve for a chosen defect is constructed.^30,31^ The resulting formation energy curve will be a piecewise linear function, with each segment corresponding to a particular slope associated with the charge that is most stable (favorable) at the given interval of the Fermi energy *E*_F_ of that segment.

An additional parameter can be extracted from eq 1 and/or from the formation energy curve directly, i.e., a thermodynamic charge transition level (the position of the Fermi energy where *q*_1_ and *q*_2_ charge states have equivalent formation energies), which can be calculated using^32^
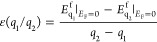
2Here,  represents the formation energy of the defect in charge state *q* considered at *E*_F_ = 0.

## 3 Results and Discussion

**Figure 1:**
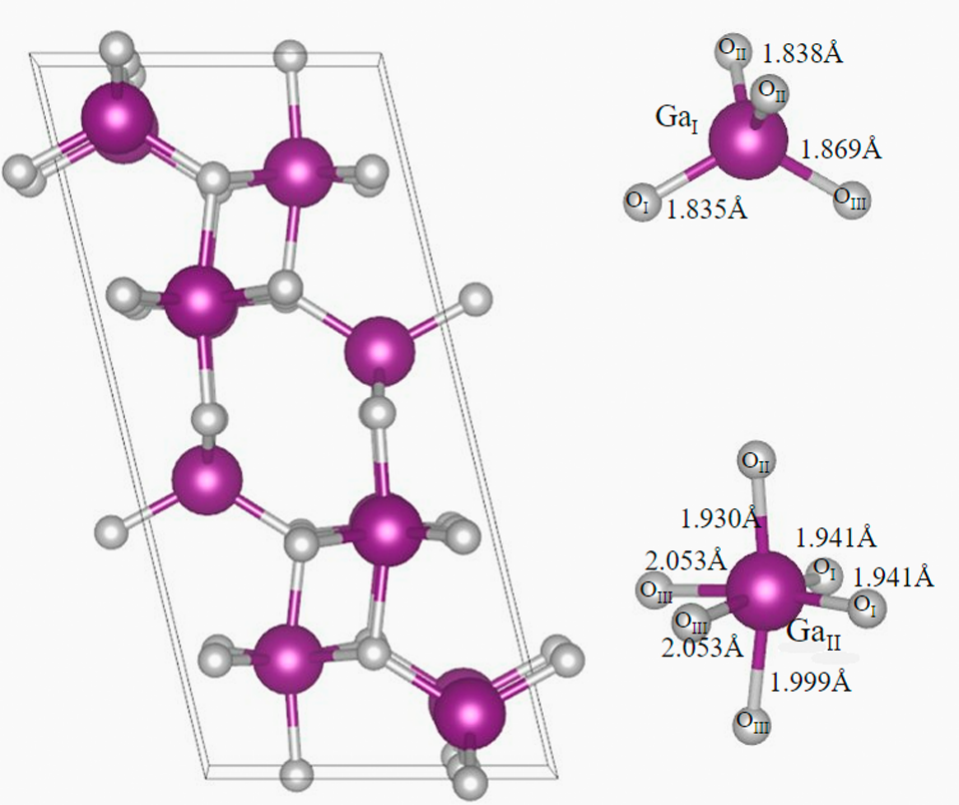
Unit cell of the monoclinic phase of Ga_2_O_3_. Coordination environment of tetrahedral Ga_I_ and octahedral Ga_II_ positions (purple spheres are Ga atoms; gray spheres are O atoms).

The monoclinic crystal structure of β-Ga_2_O_3_ belongs to the *C*2/*m* space group and consists of 20 atoms in the conventional unit cell. To determine the optimized lattice constants for the pristine β-Ga_2_O_3_ (Figure 1), energy minimization calculations were first performed for different volumes of the 20-atom unit cell. Then, optimization of the lattice constants was carried out under the constant optimized volume condition, yielding values of *a* = 12.22 Å, *b* = 3.303 Å, and *c* = 5.79 Å for pristine β-Ga_2_O_3_. These values, as well as other obtained parameters, are in good agreement with those reported in the literature obtained from *ab initio* calculations and from experiments (see Table 1).

**Table 1:** Lattice Constants, Band Gap Energy, and Formation Enthalpy of β-Ga_2_O_3_ (Δ*E*) Resulting from GGA and HSE Calculations from This Work and Compared with Experimental and Theoretical Results Found in the Literature^a^

	GGA	GGA (a)	HSE06	HSE06 (b)	exp
a (Å)	12.452	12.446	12.221	12.253	12.214 (c)
b (Å)	3.082	3.083	3.028	3.034	3.037 (c)
c (Å)	5.876	5.876	5.792	5.789	5.798 (c)
β (deg)	103.68	103.70	103.79	103.80	103.83 (c)
*E*_g_ (eV)	2.05	2.0	4.73	4.7	4.9 (d)
Δ*E* (eV)	–9.43	–9.3	–12.23	–10.3	–11.3 (e)

a note: (a) ref (33) (b) ref (34) (c) ref (35) (d) ref (36) (e) ref (35).

For the calculation of formation energies of interstitial and substitutional defects, a 160-atoms 1 × 4 × 2 supercell was created that has a size of *a* = 12.22 Å, *b* = 12.22 Å, and *c* = 11.58 Å. The choice of such a supercell guaranteed that the average interdefect distance is large enough to ensure that the error in the formation energy is small and, in addition, that this distance is approximately the same in all directions to avoid any unnecessary bias during relaxation.^14,33^ An energy cutoff of 510 eV was chosen along with a 2 × 8 × 4 Γ-centered Monkhorst–Pack^37^*k*-point grid for the unit cell. For computations on the supercell, single Γ-point calculations were performed for all interstitial cases, and a 2 × 2 × 2 Γ-centered Monkhorst–Pack *k*-point grid was used for calculations on a few selected cases. The convergence criteria were set so that the forces on each ion were less than 0.03 eV Å^–1^ and the total energy changes were reduced to less than 1 × 10^–4^ eV per atom.

As was mentioned above, the crystal structure of β-Ga_2_O_3_ comprises two inequivalent Ga sites (Ga_I_ and Ga_II_) and three inequivalent oxygen sites (O_I_, O_II_, and O_III_). Figure 1 illustrates the coordination environment of these atoms, where Ga_I_ and Ga_II_ are coordinated by four (tetrahedral) and six (octahedral) oxygen atoms, respectively. Note that there are three inequivalent positions for oxygen: O_I_ is coordinated by two Ga_II_ and one Ga_I_, and O_II_ is coordinated by two Ga_I_ and one Ga_II_, while O_III_ is coordinated by four atoms. The bond lengths between different atoms are also depicted in Figure 1.

Altogether, there were eight different initial positions, denoted as i_1_, i_2_, i_3_, i_4_, i_5_, i_6_, i_8_, and i_9_, considered for the initial position of interstitial Si atoms (see Supporting Information for a graphical visualization of these eight initial positions used in interstitial calculations). Here, the notation from ref (20) is used to identify the interstitial positions. Note that the initial position i_7_, as well as positions i_10_ and i_11_ were excluded from consideration as they were shown to be energetically unstable.^20^ The systems with doped atoms in these eight positions were then relaxed to determine the most stable configuration for interstitial Si atoms in their neutral states in the crystal lattice. Then, using a single Γ-point for a *k*-point grid, the formation energies of neutral Si interstitials were computed to identify the most promising cases of interstitial defects for further analysis. Table 2 shows that the formation energies for the Si interstitial at three positions, i_8_, i_9_, and i_5_, are close to each other and have values that are much lower than the values for other cases. These three positions, i_8_, i_9_, and i_5_, were selected for further analysis with more accurate computations (denser *k*-point grid) and with charged defects. The charge of the supercell was set to take the values 0, +1, +2, +3, and +4 for each defect in these cases.

**Table 2:** Formation Energy *E*^f^ of the Neutral (*q* = 0) Si Interstitial at Different Locations, Sorted in Ascending Order^a^

	*E*^f^ (eV)
Si_i8_	4.74
Si_i9_	5.00
Si_i5_	5.30
Si_i1_	8.28
Si_i4_	9.01
Si_i2_	9.40
Si_i3_	10.37
Si_i6_	15.10

a Note that the calculations were performed on a large supercell but with a single Γ-point as *k*-point grid.

**Figure 2:**
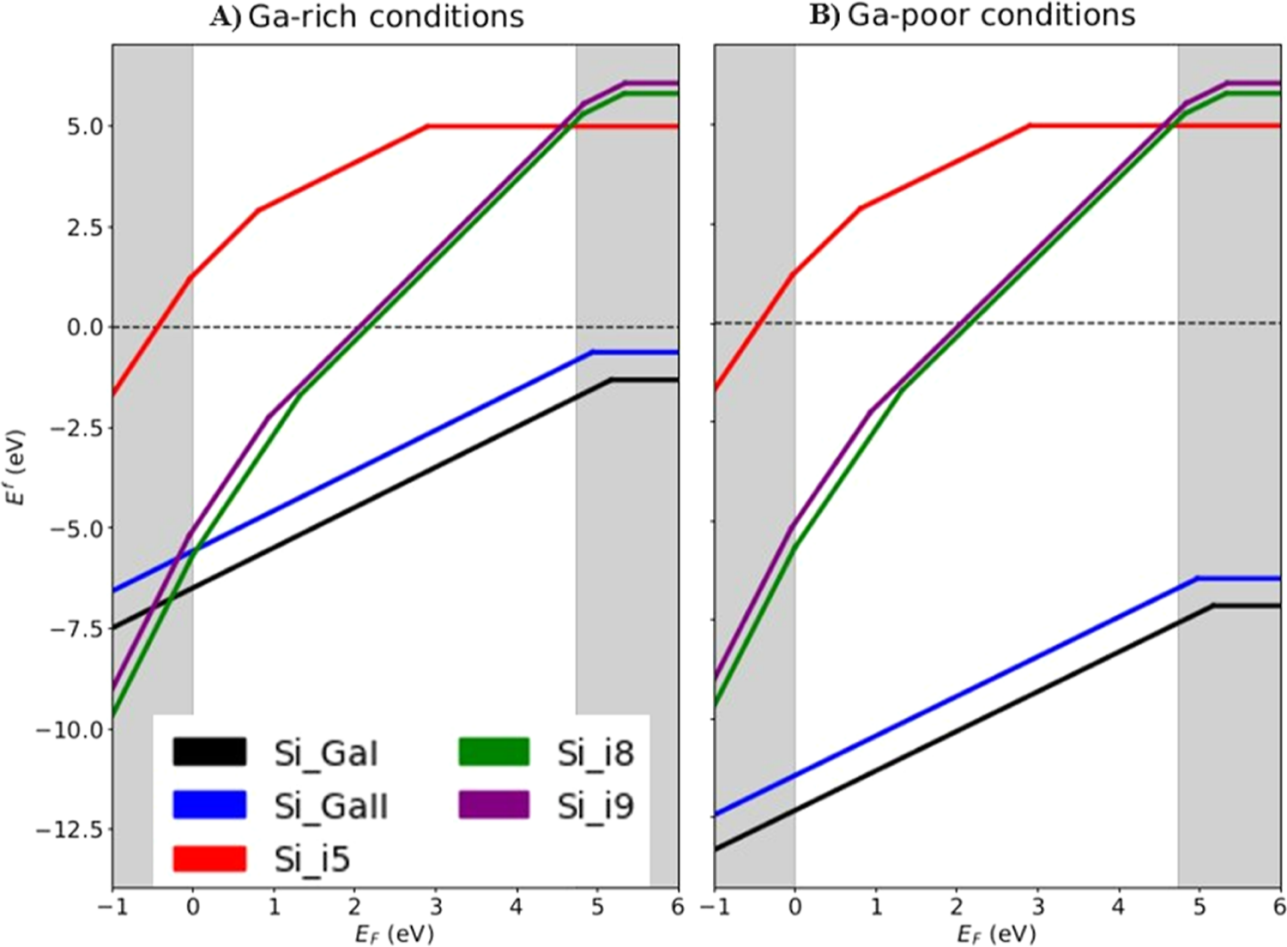
Formation energies for Si impurity in β-Ga_2_O_3_ plotted against the Fermi energy for (A) Ga-rich and (B) Ga-poor conditions. The boundaries for the nonshaded region correspond to the VBM (*E*_F_ = 0 eV) and the CBM (*E*_F_ = *E*_g_ = 4.73 eV). Note that the calculations were performed on a large supercell with a dense *k*-point grid.

Let us start the discussion with the formation energy results. As shown in Figure 2, Si substitution at the Ga_I_ site is energetically more favorable than that at the Ga_II_. For both defects, the charge state +1 is the stable one. The obtained results agree well with the literature data.^16^ Slight deviations in numerical values are attributed to the different functionals used (HSE06 hybrid functional^25,26^ in this work vs PBE0 hybrid functional^38,39^ in^16^) as well as to the different supercells used (160 atoms in this work vs 120 atoms in ref (16)). For the considered host matrix, the substitutional Si_Ga_ defects at the Ga_I_ and Ga_II_ sites are energetically more favorable than the interstitial Si_*i*_ defects over the whole band gap range and for both Ga-rich and Ga-poor conditions. Only in the case of Ga-rich conditions and for Fermi energies very close to the VBM, the formation energy of the interstitial Si at position i_8_ is slightly lower than the value for the substitutional Si_Ga_ at the Ga_II_ site.

For Si placed at interstitial positions, our calculations reveal that for all defects studied, the dependence of the formation energy on the Fermi energy is not monotonic, i.e., their charge state changes depending on the current level of the Fermi energy. For example, the charge state +3 is the favorable state for Si placed at the interstitial positions i8 from the VBM to 1.32 eV, and after that the charge state +2 is the favorable state. A similar dependence, with a similar value of the transition level, is observed for Si placed at interstitial positions i_9_. In case Si placed at the interstitial position i_5_, the neutral state could also be favorable for the higher *E*_F_ values. Overall, Si placed at interstitial positions i_8_ and i_9_ is more energetically favorable than Si placed at interstitial position i_5_ over the whole band gap range. Only for Fermi energies very close to the CBM, the formation energy of the interstitial Si at the position i_5_ is lower than for the interstitial Si at positions i_8_ and i_9_; hence, the Si_i5_ case is more favorable. Table 3 indicates the thermodynamic energy transition between different charge states for each considered defect. It is also worth noting that all considered structures correspond to positive charge states in the energy band gap region (and neutral for Si_i5_ at higher *E*_F_ values), indicating that these interstitial defects could only act as donors.

**Table 3:**
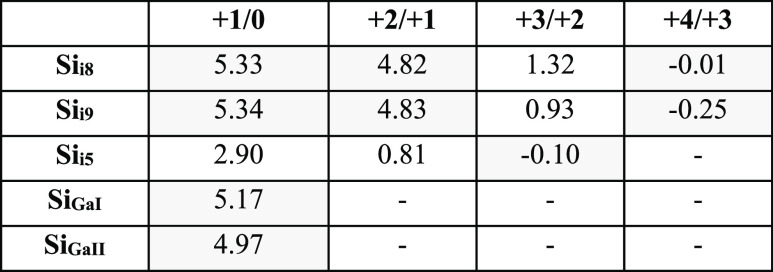
Transition Level ε(*q*_1_/*q*_2_) for Si-Doped β-Ga_2_O_3_ in the Range of *E*_F_ is Presented in Figure 2^a^

a The shaded cells indicate values outside of the band gap range (0 ≤ *E*_F_ ≤ *E*_g_ = 4.73 eV). Missing values denote transitions absent within the range of *E*_F_ presented in Figure 2 (−1 ≤ *E*_F_ ≤ 6 eV).

To the best of our knowledge, there were only a few theoretical studies on Si interstitial in β-Ga_2_O_3_,^16,18,20^ with only one study that looked into different charge states of Si interstitial.^16^ Qualitatively, the formation energy curves for Si placed at the interstitial positions i_8_ and i_9_ are similar to the curve for Si interstitial presented in ref (16) (see Figure 1 therein): the formation energy changes from the value of −9 eV at the VBM to the value + 5 eV at the CBM, crossing *E*^F^ = 0 eV somewhere in the middle of the band gap, and having stable charges +1, +2, and +3. Quantitatively, however, there are changes due to differences in the values of the transition levels +3/+2 and +2/+1, which are attributed to different functionals and supercells used as discussed earlier.

Finally, note also that the formation energy values of neutral defects calculated using a denser *k*-point grid differ significantly from those calculated using only a single Γ-point. It can also be seen from Table 2 and Figure 2 that even the order of the values is different for Si_i5_, Si_i8_, and Si_i9_. This confirms the fact that DFT calculations should be carefully checked not only with respect to the supercell size but also with respect to the *k*-point mesh. One should also keep in mind that, for a chosen Si interstitial case, the relaxation is performed for the neutral state only in this work. The resulting relaxed structures were then used as fixed structures for computations with charged defects. This is a standard approach where it is assumed that the doping procedure or any subsequent treatment steps do not allow for the host matrix to be further relaxed. However, in the future, depending on the conditions, a relaxation procedure may also need to be performed for charged defects.

**Figure 3:**
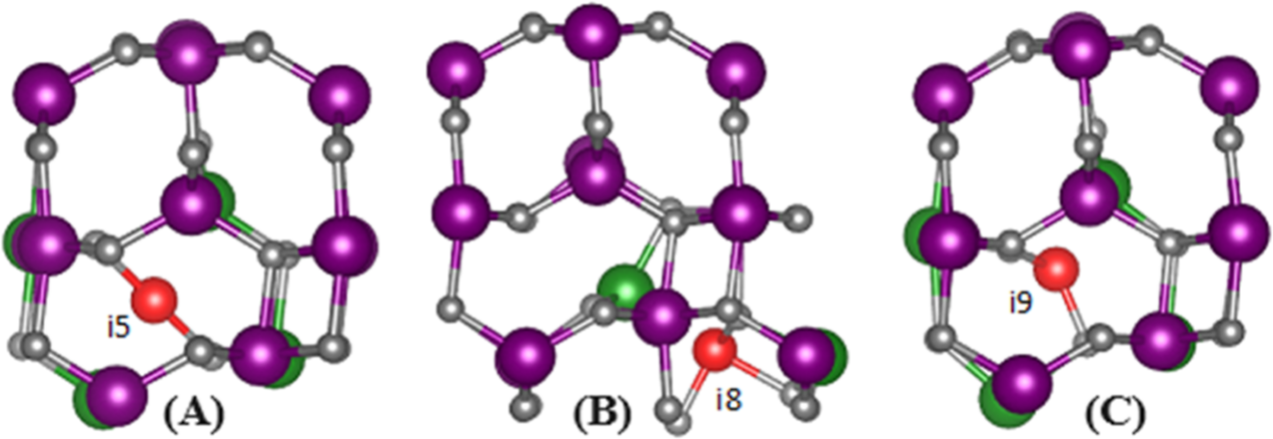
Positions of atoms after relaxation for doped β-Ga_2_O_3_ with Si interstitial at positions (A) i_5_, (B) i_8_, and (C) i_9_. Note: The red spheres denote Si atoms, gray spheres oxygen (O) atoms, purple spheres gallium (Ga) atoms, and green spheres indicate the Ga atoms shifted most substantially during structural relaxation.

For all of the studied cases, it was found that the interstitial defects led to some structural reorganization through displacing neighboring O and Ga atoms out of their initial sites. Figure 3 demonstrates the final position of the interstitial atoms and their neighbors for the cases i_5_, i_8_, and i_9_. The case i_5_ showed the least change in the initial structure after relaxation. In this case, the Si interstitial did not move from its initial position, and the neighboring atoms of O and Ga were displaced by a maximum of 0.30 Å. In the case i_8_, the Si interstitial moved from its initial position by 0.35 Å; however, one of the atoms of Ga was displaced by 0.92 Å with the rest of the neighboring atoms of O and Ga by a maximum of 0.35 Å. Finally, in the case i_9_, the Si interstitial was displaced by 0.60 Å from its initial position, which is the largest displacement of Si interstitial among the studied cases. The neighboring atoms of O and Ga were also displaced by a maximum of 0.30 Å, as in the case of i_5_. Some of the coordinates and distances for these cases are given in Table S3 in the Supporting Information.

**Figure 4:**
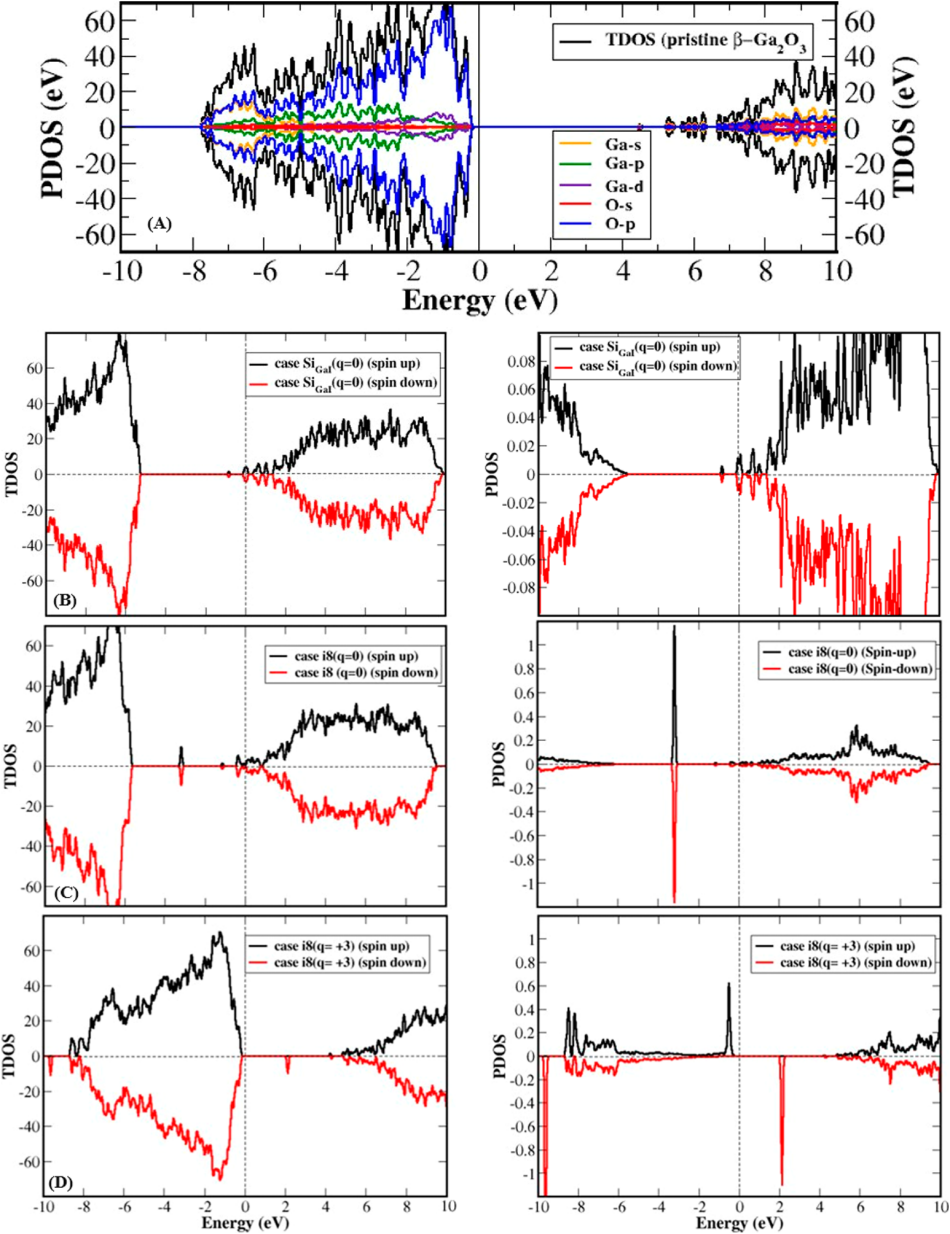
TDOS and PDOS for the cases of (A) pristine β-Ga_2_O_3_, (B) substitutional Si at the Ga_I_ site, (C) neutral Si interstitial at the i_8_ position, and (D) +3 charge state Si interstitial at the i_8_ position (the Fermi energy is set to 0 eV).

The calculated density of states (DOS) for the pristine sample, that is, the intrinsic β-Ga_2_O_3_, is shown in Figure 4A, with a band gap of 4.73 eV. The VBM clearly corresponds mainly to the O 2p states. The substitution of Si at the Ga_I_ site (Si_GaI_), which is the most favorable of the two substitution cases, causes the material to exhibit metallic behavior, as shown in Figure 4B. Figure 4C,D presents the total DOSs (TDOS) and partial DOSs (PDOS) for Si interstitial at position i_8_ with charge states +2 and +3. This choice is based on the fact that case i_8_ has the lowest formation energy over almost the entire Fermi energy range from the CBM to the VBM, and charge states +2 and +3 are the most stable (favorable) states near the CBM and the VBM, respectively. The TDOS and PDOS for cases i_9_ and i_5_ are provided in Supporting Information. Near the VBM, for interstitials at positions i_8_ (+3 charge), i_9_ (+3 charge), and i_5_ (+2 charge), the system remains a semiconductor with *E*_g_ of 2.076, 1.780, and 1.191 eV, respectively. Near the CBM, for interstitials at positions i_8_ (+2 charge), i_9_ (+2 charge), and i_5_ (neutral), the system is also a semiconductor with *E*_g_ of 2.24, 2.44, and 1.61 eV, respectively. Finally, it is worth mentioning that the charge states induce magnetic moments of 1.00 μ_B_ for the cases i_8_ (+3 charge) and i_9_ (+3 charge), 2.00 μ_B_ for the case i_5_ (+2 charge) but 0.00 μ_B_ for the case i_8_ (+2 charge) and i_9_ (+2 charge). The magnetic moment is absent for the case i_5_ (neutral). The appearance of magnetic moment can be attributed to the displacement of Ga neighbors, as previously demonstrated in a study by Yang et al.,^40^ which showed that vacancies of Ga_I_ and Ga_II_ can induce magnetism in the monoclinic phase of Ga_2_O_3_.

## 4 Conclusions

In this work, we utilized a DFT approach with a hybrid functional to study various point defects, such as interstitial and substitutional Si, in the low-symmetric monoclinic phase of Ga_2_O_3_. Our results indicate that in the case of substituting, the Si atom mostly prefers the Ga_I_ site that agrees well with the literature. In the case of eight different interstitial Si point defects studied here, first, the cases i_8_, i_9_, and i_5_ were selected based on their lower formation energy of defects at neutral charge computed at Γ-point only. Then, these cases were analyzed at different charges, selecting the most favorable among them. We found that among the studied interstitial defects, the case with Si positioned at i_8_ is the most favorable over the entire Fermi level range but could be at different charge states: +3 near the VBM and +2 near the CBM. In addition, our results show that these defects at the charge state induce a noticeable spin polarization, keeping semiconducting properties.

## Data Availability

The data that support the findings of this study are publicly available at 10.18150/1MQF0J.

## Supporting Information Available

Crystal structure of β-Ga_2_O_3_ with marked different initial positions for interstitial atoms; TDOS and PDOS for doped β-Ga_2_O_3_ with neutral and charged interstitial defects for different cases; and initial and final after-relaxation procedure positions of the atoms with the largest displacement for different cases (PDF)
