# Supplementary material for: Point Defects in Silicon-Doped β-Ga2O3: Hybrid-DFT Calculations
Source: ACS Omega. 2023 Nov 9;8(46):43732–8. doi: 10.1021/acsomega.3c05557 (PMC10666250; doi:10.1021/acsomega.3c05557)
Supplement: Supplementary file 1 — ao3c05557_si_001.pdf [file ao3c05557_si_001.pdf]

Supporting Information file for:

Point defects in silicon doped *β*-Ga_2O_3: hybrid-DFT calculations

Asiyeh Shokri[1*], Yevgen Melikhov[2], Yevgen Syryanyy[1,3], Iraida N. Demchenko[1]

1. Institute of Plasma Physics and Laser Microfusion, ul. Hery 23, 01-497 Warsaw, Poland

2. Institute of Fundamental Technological Research Polish Academy of Sciences, ul. Pawinskiego 5b, 02-106 Warsaw, Poland

3. Institute of Microelectronics and Optoelectronics, Warsaw University of Technology, ul. Koszykowa 75, 00-662 Warsaw, Poland

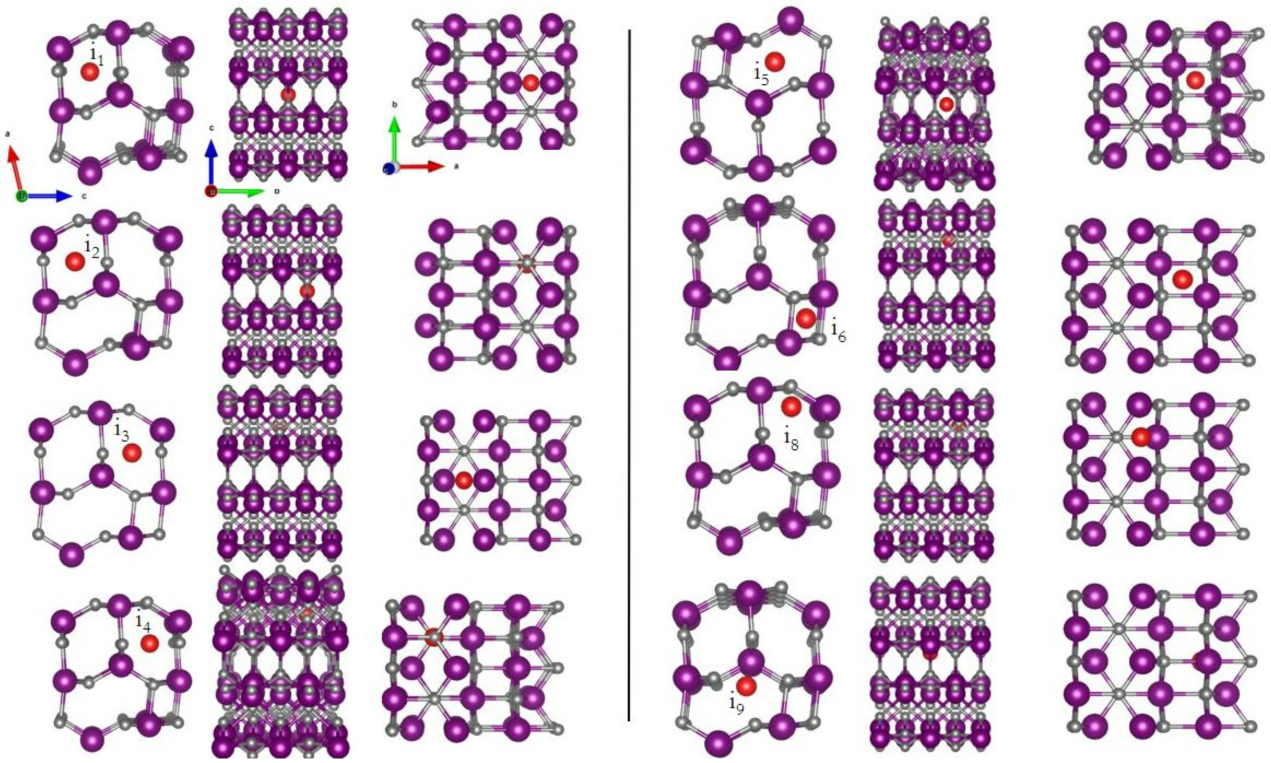

Figure S1: Eight different *initial* locations associated with the interstitial positions of *β*-Ga_2O_3 structure (top and side views). Note that red spheres represent interstitial atoms (Si in our case), violet spheres are atoms of Ga and grey spheres are atoms of O.

[*]Corresponding author: asiye.shokri@gmail.com

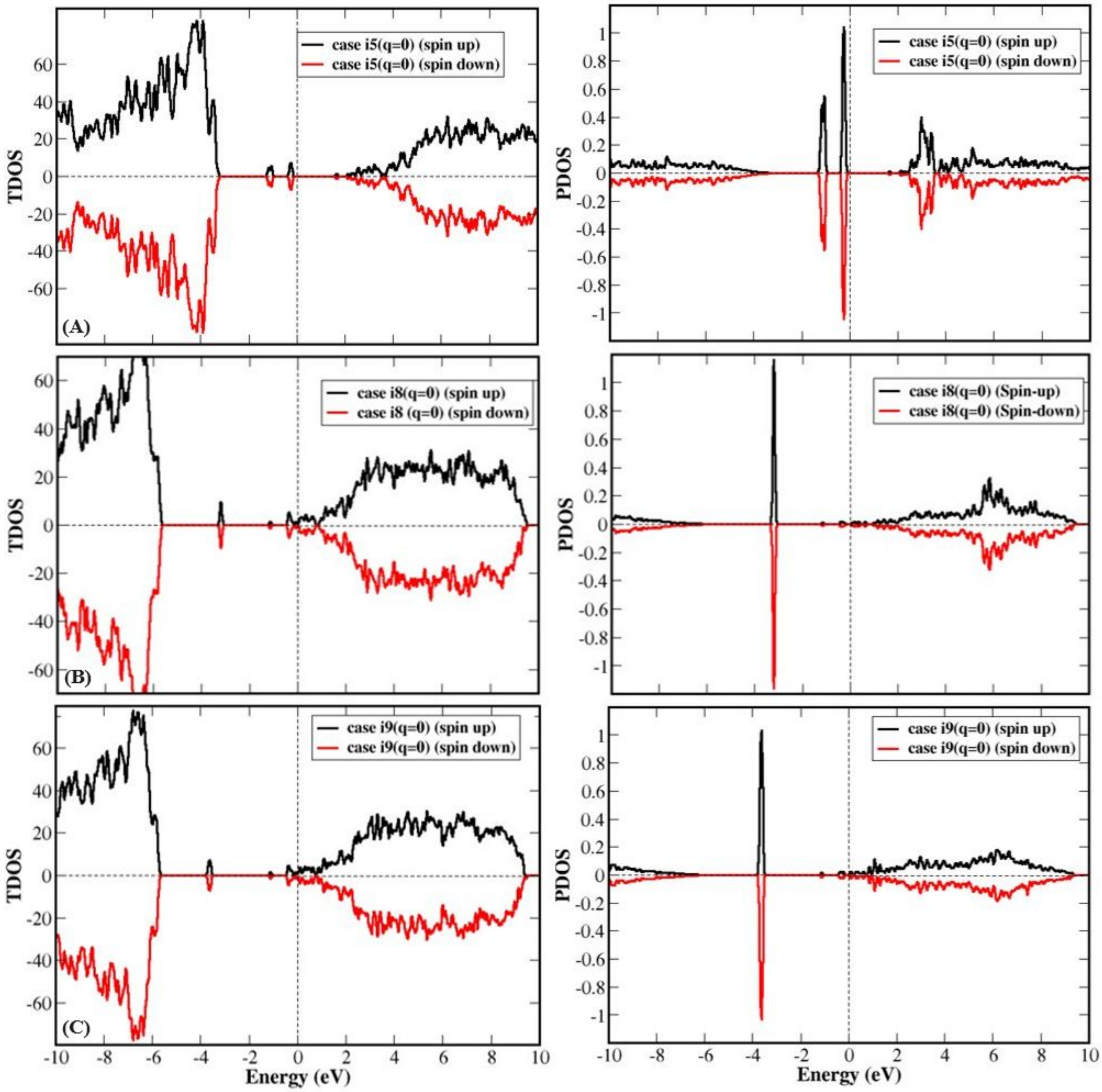

Figure S2: Total density of states (TDOS, left column) and partial density of states (PDOS, right column) for doped β-Ga_2O_3 with *neutral* Si interstitial at (A) i_5, (B) i_8 and (C) i_9 positions, respectively (The Fermi energy is set to 0 eV).

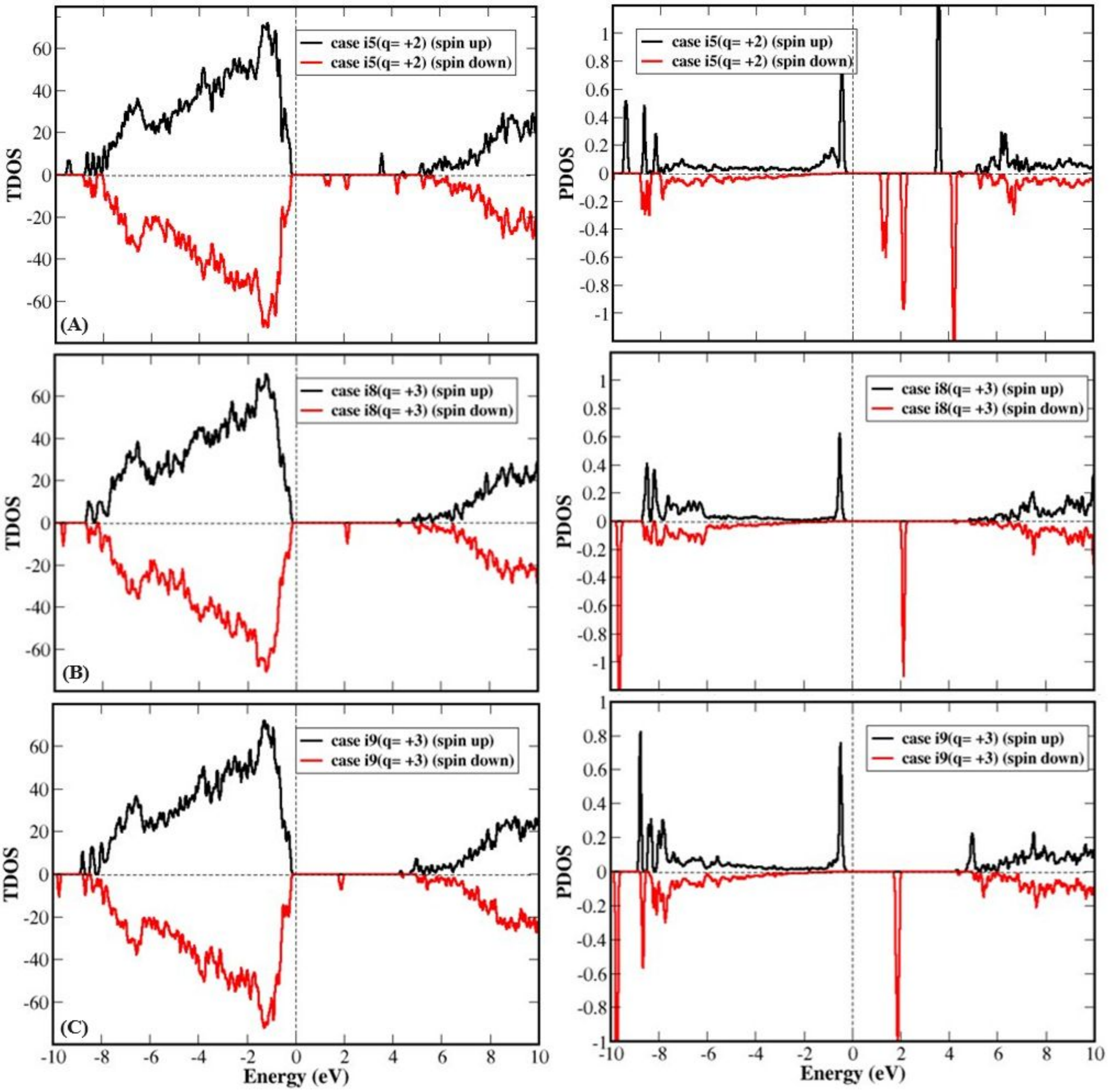

Figure S3: Total density of states (TDOS, left column) and partial density of states (PDOS, right column) for doped β-Ga_2O_3 with *charged* Si interstitial at (A) i_5 with charge +2 (B) i_8 with charge +3 and (C) i_9 with charge +3, respectively (The Fermi energy is set to 0 eV).

Table S1: Initial and final after relaxation procedure positions of the atoms with the largest displacement for the cases i_5, i_8 and i_9.

		Initial position			Final position			Distance from Si atom, Å	Distance displaced during relaxation, Å
**i5**	Si	0.2500	0.3125	0.5000	0.2500	0.3125	0.5000	0.0000	0.00
	O99	0.3347	0.3750	0.4444	0.3428	0.3745	0.4255	1.7500	0.26
	Ga19	0.0910	0.2500	0.3979	0.0848	0.2606	0.3735	2.2821	0.30
	O97	0.3347	0.1250	0.4444	0.3313	0.1345	0.4419	2.5316	0.12
	Ga11	0.3409	0.2500	0.3416	0.3455	0.2477	0.3199	2.7373	0.27
	O83	0.1734	0.2500	0.2811	0.1735	0.2451	0.2587	2.8477	0.27
**i8**	Si	0.098	0.3750	0.7660	0.1011	0.3750	0.7371	0.0000	0.35
	O116	0.9969	0.3750	0.6283	0.9746	0.3750	0.6392	1.6859	0.33
	O84	0.1734	0.2500	0.7811	0.1601	0.2495	0.7929	1.7400	0.27
	O76	0.0031	0.3750	0.8717	0.0239	0.3750	0.8583	1.8692	0.33
	Ga4	0.1591	0.3750	0.6584	0.2023	0.3750	0.6029	2.2059	0.93
	Ga20	0.0910	0.2500	0.8979	0.0885	0.2389	0.9200	2.7178	0.30
**i9**	Si	0.3170	0.2500	0.5605	0.2864	0.2500	0.5117	0.0000	0.60
	O108	0.1653	0.2500	0.5556	0.1585	0.2500	0.5302	1.6271	0.29
	Ga28	0.4091	0.3750	0.6021	0.4242	0.3664	0.6281	2.3577	0.33
	Ga26	0.4091	0.1250	0.6021	0.4241	0.1336	0.6281	2.3577	0.33
	O90	0.3266	0.1250	0.7189	0.3253	0.1203	0.7287	2.9048	0.13
	O92	0.3266	0.3750	0.7189	0.3253	0.3797	0.7286	2.9048	0.13
